# The Digitalization of Social Care in England and Implications for Older, Unpaid Carers: Constructionist Thematic Analysis

**DOI:** 10.2196/60056

**Published:** 2024-10-24

**Authors:** Anastasia Rousaki, Efpraxia D Zamani, Laura Sbaffi, Kate Hamblin, Rachael Black

**Affiliations:** 1 Information School University of Sheffield Sheffield United Kingdom; 2 Durham University Business School Durham United Kingdom; 3 Centre for Care University of Sheffield Sheffield United Kingdom

**Keywords:** social care, England, digitalization, digital transformation, unpaid care, mobile phone

## Abstract

**Background:**

Globally, populations are aging, generating concerns about the sustainability of health and social care provision. In terms of the public provision of social care in particular, unpaid carers provide much of the support to people with disabilities and older people. In addition, there is an increased onus in many countries on digital transformation projects, in the hope that the digitalization of services can create efficiencies and savings in both costs and care labor. In England, the focus of this paper, the shift to digital services is also framed as a means to enhance choice and control for older unpaid carers, while being part of a broader offering that includes nondigital alternatives and support to mitigate digital exclusion.

**Objective:**

This study examines the impact of digitalization on older, unpaid carers—a group more likely to be both expected to engage digitally with services and at risk of digital exclusion—in England, focusing on their lived experiences in terms of caring and access to social care.

**Methods:**

We used a constructionist approach to thematic analysis, where data from 48 older unpaid carers collected through focus groups were analyzed using thematic analysis, resulting in 4 prevailing themes.

**Results:**

Our findings indicated that while unpaid carers largely acknowledge the benefits of digitalization, they also highlight several points of failure, whereby engagement with digital spaces is experienced as coercive and exacerbates feelings of exclusion. These are further worsened by government failures to address issues of connectivity, imposing additional financial burdens and complicating tasks such as benefit applications.

**Conclusions:**

In this study, we have highlighted the need for greater involvement in shaping both policy and technological solutions, which in turn will be more inclusive and aligned to the aspirations and circumstances of older carers.

## Introduction

There are concerns globally regarding the sustainability of health and social care systems as populations are aging and growing numbers of individuals aged ≥65 years are facing unmet care needs [1]. Unpaid or *informal* care has long been part of “care diamonds” (the configuration of care provision among states, markets, families, and the third sector [2]). Indeed, the role of unpaid care is increasing in the provision of care, as even in nations where statutory provision had been fairly comprehensive, retrenchment is “bringing the family in [to caring arrangements] through the back door” [3]. Therefore, unpaid carers provide a large majority of all care.

In the United Kingdom—and specifically England, which is the empirical focus of this paper—unpaid care is an important societal phenomenon. Census data indicate there are 5.7 million unpaid carers [4], but based on other analyses and due to challenges around self-identification, the numbers seem to be closer to 10.6 million [5]. Aging and unpaid care are closely related, with older age groups providing the highest number of hours of care [4]. Estimates also put the value of care provided by unpaid carers in England and Wales annually at £162 billion (US $211 billion), exceeding the annual expenditure on the National Health Service (NHS) [6]. However, in the past decade, there have been significant cutbacks to social care funding and provision, with such austerity measures often being underpinned by neoliberal ideologies [7]. This financialized, technology-oriented policy agenda places the onus of care provision and financing onto families and communities while limiting their capacity to provide care via the imposition of austerity [8]. This is particularly the case in the United Kingdom, with care-related austerity policies impacting mainly susceptible individuals, namely minority ethnic groups, women, and people with disabilities [7].

The combination of such austerity policies and the increased need for care has resulted in the increasing use of digital interventions in social care, where these primarily aim toward cost reduction [9]. Digitalization has now become a global phenomenon impacting both professional and personal life through the increasing prevalence of digital technologies for the development and provision of mobile health, health information systems and technologies, wearable devices, and telehealth. Within public discourse, such interventions are often promoted by key political stakeholders, who suggest that digitalization will improve care almost deterministically by creating efficiencies [10]. In relation to social care, technologies have been espoused by the Secretaries of State for Health and Social Care in England as “transformative” and as a means to create efficiencies and “free up” care labor and financial resources [11].

The digitalization of care, or the shift to the use of technologies in the provision of care including through web-based services, is also framed as a means to enhance choice and control for people in receipt of support and unpaid carers. The NHS App and the NHS website aim to “become platforms through which people, their families and unpaid carers can access more services and resources proactively have more control over their care benefit from more personalised and preventative offers” [12]. The underpinning notion is that digital options will increase both choice and allow for “informed decision-making” through access to data and a wider range of services. While policy documents also indicate that nondigital options and services are to be retained for those who cannot or do not wish to use web-based services [12], technological visions often lead to exclusions and further marginalization [13]. The reasons are diverse as several factors contribute toward digital exclusion, including gender, age, gender identity, disabilities, and ethnicity, to name only a few [14], as well as unpaid care [15]. Among these, age is a significant factor. Until recently, older adults aged ≥65 years constituted only 5.5% of the global digital population [16], and consequently, the implications of digitalizing essential tasks for older adults can be particularly pronounced: many everyday activities nowadays are either solely or primarily web-based (eg, benefits, tickets, and banking), and these are known to pose difficulties for older adults, thus further reinforcing existing exclusions [17]. In addition, digital exclusion can be accentuated through the confluence of roles and identities. For instance, older adults are more likely to be engaged in unpaid care (in 2021, two-thirds of unpaid carers were older adults) [18]. Therefore, digital exclusion of older adults, coupled with their likelihood to be unpaid carers, has spurred growing concerns [19].

Given the substantial interrelation among aging, digital exclusion, care, and socioeconomic factors [20], we argue that it is imperative to elucidate the effects of digitalization of care on older, unpaid carers. In this study, we asked, “What is the impact of the digitalization of care on older adults and particularly those with care-related responsibilities?” and built on previous work by examining unintended consequences and empirically interrogating the promissory discourse of digital health [21] and, in this case, social care.

We contextualized our study within England, United Kingdom, due to its distinctive social care landscape and the policy onus on encouraging widespread adoption of care-related technologies [1,10]. In subsequent sections, we have first provided an overview of our methods and then presented our findings in detail. This is followed by a critical analysis and discussion of our findings in relation to the digitalization of care and the connections and tensions between unpaid carers’ experiences and the prevailing political focus on digitizing social care.

## Methods

### Overview

We conducted focus groups in care centers located in Liverpool, Devon, Leeds, Derby, and Bath and virtually via Google Meets (Google LLC) between September 2023 and February 2024. The location choice constitutes a contextual exploration of the effects of digitalization in relation to the north-south divide. The divide is a postwar Britain concept, characterizing the bifurcation of the varying socioeconomic conditions across England [22], which often negatively affect the northern regions.

Focus groups allow participants to coconstruct meaning and explicate normative discourses and how inequalities are worked up via discussions [23]. These focus groups were designed with the help of story completion prompts [24] that entailed stories relating to digital poverty, digitalization policies, and web safety. To provide a sense of detachment, we used gender-neutral names and third-person story prompts.

### Recruitment

Recruitment was conducted via carer centers and our research partners. This outreach strategy involved advertising through carer centers’ mailing lists and newsletters, displaying posters in the carer centers, and promoting the research during their coffee days. Details regarding our focus group participants can be found in Table 1.

**Table 1:** Focus groups and participant information (all participants were aged ≥50 years).

Location	Participants (N=48), n (%)	Women (n=35), n (%)	Men (n=8), n (%)
Liverpool	13 (27)	9 (19)	4 (8)
Devon	14 (29)	14 (29)	0 (0)
Leeds	7 (15)	6 (13)	1 (2)
Bath	5 (10)	3 (6)	2 (4)
Derby	5 (10)	—^a^	—
Virtual focus group	4 (8)	3 (6)	1 (2)

^a^Participants preferred not to disclose their identity.

### Data Analysis

The lived experiences of care, as well as its accompanying sociocultural structures, institutions, and apparatuses, constitute a complex topic; this is particularly the case when studying it through the lens of digitalization [25]. Consequently, we used a constructionist thematic analysis that focused on the construction of ideological subjects [26,27]. We began by immersing ourselves in the data, whereby we repeatedly and iteratively reviewed the collected data to independently identify codes and themes; document our observations; and organize the data into codes, subthemes, and themes. We began coding line by line to familiarize ourselves, and we then revisited the preliminary set of codes to identify overlaps, similarities, and differences. This resulted in merging codes together and eventually forming subthemes. These were then cross-checked between members of the research team again for cohesiveness and consistency. Finally, initial themes were organized and discussed; they were then reevaluated within the entire dataset, refined, and labeled.

Throughout the analysis and write-up, we considered questions such as the implications of the themes and potential justifications of participants’ constructions within the sociopolitical context. Our analysis resulted in 4 prevalent themes, which are discussed in subsequent sections (Multimedia Appendix 1).

### Ethical Considerations

The research received ethics approval from the University of Sheffield, United Kingdom (approval 053260), and due to the sensitive nature of our research, the focus groups were not audio recorded and we kept handwritten notes. No personal information was collected from participants, and all potentially identifiable information was anonymized or removed from the data. Before the workshops, participants received a comprehensive information sheet describing the project's purpose in lay terms and signed a consent form. The consent form outlined their right to withdraw from the study at any time. Compensation was provided to each participant in the form of a £40 (US $52) shopping voucher.

## Results

### Overview

In what follows, we present the findings of our thematic analysis. In brief, our findings indicate that older, unpaid carers and people needing care perceive the digitalization of social care as an experience of them being pushed *online* (intended here as being forced to engage with the digital), without however having the required support or the provisions necessary for meaningfully interacting with the digital world. This in turn is a great digital disconnect element, whereby they feel as if the lack of connectivity and the associated costs, coupled with the digital push, requires them to negotiate such support and provisions in market (or economy) terms to make their case. Consequently, while there are benefits in the use of digital technologies in social care (eg, accessibility), there are structural challenges that prohibit the fulfillment of digitalization’s potential.

### Being “Pushed” Online

#### Overview

Carers constructed their engagement with digital technologies as compulsory and reported feeling coerced into *online* engagement, describing the experience as being “pushed” toward it. This pressure exacerbated their sense of exclusion, as viable offline alternatives or simpler systems were hard to access or completely substituted. They further described this by highlighting that their engagement with web-based care services is based on pseudoconsent, characterized by the lack of informed decision-making or alternative options. Participants advocated for personalized, human-centric approaches and preserving face-to-face interactions, particularly for future generations who may encounter similar challenges. Within this theme, we found the subthemes explored in subsequent sections.

#### Lack of Agency and Pseudoconsent

Our participants described the digitalization of care in ways that appear ideologically hegemonic [28], whereby such efforts are driven by assumptions of equal access to web-based communications. They often feel obligated to navigate digital services and the digital world in general while experiencing challenges with web-based transactions. The pressure to exclusively adopt digital processes was a common theme (eg, “I feel like I’m being pushed online”); however, participants’ constructions were in tension with such societal assumptions, prompting reflections on the absence of choice within a democratic society. In many ways, participants resisted the current status quo with remarks such as “I want people to have an alternative,” providing clear arguments as to why this was important.

They emphasized the problematic nature of the expectation to conduct all activities digitally, questioning the lack of consent regarding their involvement in digitized activities. Participants questioned the data-retention practices of companies, as well as their lengthy terms and conditions (T&Cs), with participants being uncertain about the consequences of agreeing to confusing disclaimer notices (“These documents are a legal contract, meaning they can’t get the blame for anything”). In turn, such feelings of coercion further increased, due to the sense of inevitability in agreeing to T&Cs, as failure to do so would result in the denial of essential services (“You have to agree, or you don’t get the services”). Such power imbalances were constructed as inherently related to the nature of caring; participants perceived consenting to anything as a binary, a choice between contributing to the well-being of their loved one or having to face the potential consequences of not accessing such services.

Other assumptions they challenged relate to those regarding web-based safety and security and confidentiality. Carers expressed apprehension about providing information on the web, citing unclear T&Cs and the intimidating experience of having to navigate numerous pages. They further noted that there is added complexity in comprehending content, often due to the absence of accessible language (“Deliberately written to be non-informative!”). App permissions (eg, permission to access the computer’s camera) raised further inquiries, with 1 participant querying, “Why, what else are they looking for?”

#### Maintaining Alternative Options

In contradiction to the policy statements regarding web-based services being a part of “multichannel offers” [12], participants highlighted concerns about the diminishing availability of traditional support systems, particularly noting the post–COVID-19 era’s dearth of in-person options and telephone helplines. Accessing services remotely can be challenging, as often it is impossible to contact anyone for further support. They thus resisted the transition of care-related companies to digital platforms, which necessitate web-based access and the navigation of digital apps. Instead, they highlighted that reading information on paper is more straightforward.

The importance of being able to choose and having options was framed as a matter of inclusivity (“We have to be inclusive, we can’t make people do it”), emphasizing the importance of ensuring that every organization provides alternatives. Participants suggested that having a telephone consultation in person would be helpful, as it takes the same amount of time and maintains a “human” element (“People need personalisation and a human touch and technology takes that away”).

Furthermore, carers voiced the need for a round-the-clock helpline where they can interact with a live person instead of navigating automated prompts (“I don’t want to hear horrible stupid inanimate words,” expressing a preference for speaking to real people over automated systems). Stressing the necessity for 24/7 availability, carers argued that nighttime incidents pose particular challenges, as they are characterized by limited support, increased isolation and vulnerability, and by carers being more fatigued and stressed.

#### Digital Alienation and Exclusion

Participants vividly described technology as “dangerous” and challenging (“As soon as someone tells me, ‘I send you the link,’ my heart sinks!”), in terms of contributing to their marginalization, even in seemingly positive cases (“Technology is amazing but it excludes so many people”). Feelings of alienation and a lack of support were common discussion points (eg, “nobody cares”). For example, if any information is missing, it is often considered the carers’ fault (“you feel like you are up against it all the time”). This lack of support further feeds into experiencing neglect (eg, “abandoned in the wilderness” and “who is gonna care for us”), even in cases where they need to use blood pressure monitors without assistance. Another element that contributes to feelings of alienation is when they are left facing black screens and computers (“It makes you feel more isolated”).

Feelings of exclusion were further communicated in terms of generational disparities, with reference to the unfulfilled promises of technology. Society is perceived as depriving older individuals of the choice to engage with technology at their own pace or to learn according to their preferences (“Society is not allowing older people to have a choice regarding using technology or to learn at their own pace”), which is interpreted as abandonment and lack of caring (“Nobody cares about me”; “People are not kind to older people”). Participants felt like outsiders compared with technologically proficient younger generations, citing anxiety caused by technological complexity. For example, while talking about the rapid pace of technological advancement, 1 participant constructed their predicament by using an example of a computer purchased just a few years ago that is already deemed “vintage” and said that “you are fighting the up-to-date-ness,” emphasizing the struggle to stay current.

### Supporting Carers to “Digitalize”

#### Overview

Carers highlighted the lack of assistance to access digital and web-based support as a barrier to the shift toward “digitalization.” In their discussions, they stressed the pervasive requirement for assistance in their everyday lives and juxtaposed this against the lack of formal instruction in navigating web-based systems and training (eg, in terms of privacy and safety), which could alleviate some of their fears regarding data security. They constructed digitalization as inherently ableist and advocated regarding addressing ableism and accessibility concerns via inclusive policy initiatives and the development of tailored assistive technologies.

#### Skills Gap and Training Needs

Pushing individuals toward digital services without providing guidance was heavily critiqued. Participants highlighted the lack of accessible resources for queries, using rhetorical questions such as “Am I doing this right?” with humor. They reported poor explanations in instructional videos, making it challenging for those without internet access. The public library, once a place for help, is no longer reliable due to cutbacks. Similar concerns were raised about general practitioner (GP) surgeries. In relation to this, participants resisted the notion of digitalization as “common sense” by challenging the occasional supply of free devices, stating that it does not tackle the need for assistance to use devices in practice.

Further reflecting on the nature of their personal circumstances, participants framed digitalization as social exclusion toward older carers. Indeed, the need for assistance was omnipresent in various aspects of daily life, including banking, health care, and other services. Participants expressed concern about the diminishing availability of in-person help compared with the pre–COVID-19 era and emphasized the need for training in the broader community and among care-related professionals, advocating for community-based training because, while support may be available, accessing it requires either using the internet or commuting. Some of their suggestions included drop-in centers for carers to seek assistance, emphasizing the urgency for policies addressing the assistance gap, possibly with tailored training modules and continuous support available.

#### Concerns Regarding Privacy and Surveillance

One of the major areas in which participants indicated the need for support and training is that of internet safety and privacy, as the digitalization of essential services brings forward concerns regarding data (eg, “My safety is at risk”), which can be particularly problematic when one is not aware of how their data are handled (“Unknown lurking behind the door”).

These concerns were raised as part of broader skepticism toward emerging technologies. Participants constructed a sense of panoptical surveillance, where their data and information can be easily accessible, leading to concerns and constructions about the private domain becoming public (“Nothing is accurate or private; everything is virtual and everything is fake”; “I find that worrying, that anybody can tap into what you are saying in your own home”; “Hospital at homes...everything to do with your treatment is public knowledge”). This sense of panoptical surveillance was described as a contemporary societal phenomenon (eg, “...trivialised our generation...we would never go around announcing things to 100 people...telling people all our personal details”).

Data protection is thus a highly sensitive topic, giving rise to questions regarding the security of technologies used in social care (“How secure is all of this?” “How many people read that?” and “I don’t want my stuff on there”). Yet, such perspectives are further influenced by a lack of understanding regarding the purposes of data collection and use (“People are worried about where data go and what happens to their information”; “we don’t know what is happening to this information”). Considering the domain of social care, which often intersects with health care, carers are concerned that while technology is used for efficiency, providers (including the British NHS) are still susceptible to cybersecurity issues (“I get worried because things get lost, all records can go missing”; “I worry about being hacked”). Such concerns are not unsubstantiated: multiple participants shared personal experiences of losing money and falling victim to internet scams or struggling to identify which web-based activities were scams and which were safe (“I got scammed last Friday...they know more about me than I do”). In this context, technology designers and policy makers were framed as indifferent to issues of cybersecurity and safety in the name of profit (“There’s obviously a flaw, should have been to policy makers before they released that”; “Designers are here to make money; they don’t care for you”).

#### Issues of Ableism and Accessibility

Participants emphasized the need to address ableism. They often reported that the person they care for needs help with technology or with daily tasks in general. Most often, carers themselves live with disabilities, making it difficult for them to use technology, and they emphasized the need for more accessible devices (“Access is important...we need accessible things because it is good stuff, but what’s the point if it’s not accessible?”). Participants shared their struggles with concentration issues, needing multiple screens, and facing memory or vision problems when accessing web-based tasks (“You are given something you forget how to do, and you feel embarrassed”). These aspects are exacerbated by the different conditions older adults often experience. For example, a participant with hearing loss reported experiencing diminished communication abilities and a consequent fear of being perceived as less intelligent than in the past. This can have a profound effect, as when alternative support is available, it is typically via customer support–automated call centers that one must navigate before connecting to a human agent.

Crucially, and throughout the focus groups, carers constructed digitalization as ideologically hegemonic and expressed concerns about its abruptness. They resisted this technological prevalence by questioning the societal assumption that everyone has knowledge of digital technologies and that everyone should be actively using the internet (eg, “I felt very guilty and I still do” for lacking technical knowledge).

### The Great Digital Disconnect

#### Overview

The “great digital disconnect” refers to the failure of digital services and processes to address digital poverty, a situation where the individual is unable to interact with the digital world in full when, where, and how they want or need to [14]. As participants were older adults, most of whom are state pension recipients, digital poverty issues pose additional financial strain. For them, this lack of support is counterintuitive because supporting unpaid carers could ultimately benefit the state. This in turn suggests that they feel the need to construct their labor within a neoliberal discursive framework to legitimize their need for financial support.

#### Lack of Connectivity

Concerns surfaced regarding the dearth of connectivity, resulting in extensive time spent on paperwork due to persistent connectivity issues. The shift from traditional landlines to digital platforms further heightened participants’ apprehensions, more pronounced among participants from the Southern and Midland regions of England and particularly those residing in rural settings.

The repercussions of inadequate connectivity impacted the material circumstances of the participants, with some resorting to significant expenditures for connectivity via their mobile phones. Carers depicted the lack of connectivity as frustrating (“I tried that 4-5 times, and I rang because it wasn’t working...I gave up on it”), exacerbating financial or other care-related challenges, such as benefit or grant applications disappearing without an option to save progress (“it disappears, and you have to start all over again”). This deficiency in connectivity was portrayed as compounding the shortage of time (“What should take you one hour takes you all day”), a perennial issue faced by carers, and was construed as mentally taxing, adding to the “invisible” labor already shouldered by carers.

Throughout our discussions, participants consistently raised concerns regarding the impending transition to an all-digital landscape. The prevailing sentiment was one of unease concerning the growing dependence on digital communications, underpinned by scenarios involving power outages, prompting questions about the reliability of digital systems during electricity disruptions. The consequences of increased technological reliance are even more threatening, whereby a lack of connectivity may result in irreversible failures (eg, for individuals dependent on medical devices).

In many cases, participants indicated that internet providers have increased responsibility in addressing some of their connectivity issues. As explained earlier, slow internet speeds impede access to essential services, which can be even more significant for those benefiting from social tariffs, as connections are described as notably “sluggish.”

#### Cost of Connectivity

Participants described navigating the financial aspects of technology and internet-related expenses as a “nightmare.” The cost of broadband services was labeled as a “cutthroat business,” with participants expressing a lack of awareness regarding variations in costs and services, pointing out the financial strain on their state pension. One participant noted that, with providers, the internet is bundled with other services, limiting their ability to shop around. They stressed the importance of balancing internet costs with desired services, stating, “You get to balance what you pay for the internet against what you want to have.”

Technology was described as “fancy ideas,” but participants emphasized the need for significant financial resources, stating that “there is a need for lots of money for crap.” The discussion revolved around laptop prices and the increased cost of owning one. The cumulative costs of technology, such as electricity to power a laptop, were also mentioned, which is disadvantageous to those who may not fully understand these expenses.

Affordability concerns led some participants to limit internet use to their homes, avoiding data use outside. Pay-as-you-go options were preferred, with some participants lacking awareness of potential savings when purchasing a dedicated phone for internet use. A participant shared the financial strain caused by background apps “eating away” at phone credit, despite infrequent phone use. Carers highlighted the need for financial assistance and education on cost-effective technology use.

Participants found it challenging that the devices often needed to be up-to-date to perform certain tasks. For instance, an iPhone (Apple Inc) was mentioned as necessary for diabetes-related devices and apps, but it had to be the latest version to use the required app. However, carers suggested they were not concerned about having the latest technology; thus, such demands led them to spend money updating their phones. Moreover, participants highlighted that apps often charge for access, adding to the overall cost, and emphasized the need for funding for computers.

Some participants noted that social tariffs were available only to those on benefits, leaving others to cover all expenses themselves and potentially incur debt. The perceived inadequacy of social tariffs, with slow internet speeds not justifying the cost, was raised. Applying for Carers’ Allowance requires internet access and a printer, creating barriers. Navigating available entitlements and benefits was described as a “nightmare.” Carer Allowance can be claimed until pension age, creating additional financial challenges as pensions are considered income, yet carers suggested they need the allowance on top of that.

#### Mobilizing Neoliberal Ideals and Mirroring Neoliberal Discourses to Leverage Support

Participants brought up concerns regarding devices and overall funding, not just for training but also for health care and digital inclusion. They discursively resisted and challenged the ideologically hegemonic role of technology, emphasizing that not everyone possesses computers or phones nor can they afford broadband. Participants discussed that funders should be aware that relying solely on basic pension retention may only cover essential needs such as food, leaving individuals unable to afford other necessities. Therefore, they emphasized the importance of making technology accessible, suggesting that refurbished devices could be distributed through carers’ trusts or voluntary organizations, saying, “What do you do about that...tablets, free SIM cards, other things.” In addition, carers suggested allocating funds within organizations for community services specifically targeting digital exclusion, such as providing devices, data, and staffing, saying, “Give them money, give them laptops.”

The need for funding was also discussed in the context of training. Participants proposed that training requires financial support and assistance to individuals requiring help with equipment and access. They used inclusive rhetoric via stressing the importance of viewing each person as an individual, stating that maintaining someone’s well-being comes with significant costs. They often suggested that not only volunteers but also paid workers could play a role in providing technological training and support.

Against this background, it is of analytic interest that carers negotiate that they are worthy of funding and technological inclusivity by framing their value in market terms, often discussing the financial surplus they create through their labor and the social care expenditure the government avoids as a result of unpaid care (“Carers save millions for the government”; “we save the government millions of pounds...the stress we are under, anxiety”). In many ways, carers constructed themselves as the idealized neoliberal individual, not due to wanting to be one but to legitimize their need to be digitally included (“in the end, the more savvy we are, the more money we save”), perhaps pronouncing the ideological hegemony of neoliberal constructions. Indeed, a lack of funding can impact health care and, in turn, the overall economy (“funding has to be brought somewhere otherwise everything will cost more because our health will be worse”).

### The Unfulfilled Potential of Digitalization

#### Overview

This theme concerned the unfulfilled potential of digitalization. Participants in the study acknowledged the numerous benefits of technology yet frequently framed digitalization as a promise that remained unfulfilled, expressing disappointment in the unrealized potential of technology. Technological innovations were perceived as overlooking their own requirements and lacked lived experiences. Participants raised the necessity for greater agency in informing the design of technological solutions to ensure that the potential for technology to aid and facilitate materializes. They also expressed a desire for increased agency in shaping policies and technologies aimed at carers, emphasizing the importance of research informed by lived experiences and coproduction methods. This theme resulted in numerous subthemes, as outlined in subsequent sections.

#### Benefits of Technology and the Unfulfilled Potential of Digitalization

Despite numerous challenges, participants expressed a belief in the potential of technology to enhance their lives. Some adopted an optimistic perspective, encouraging others to persevere, asserting that embracing technology will eventually lead to a better quality of life for those who are willing. Drawing parallels to historical revolutions that simplified various aspects of life, participants pointed to the ongoing digitalization as a comparable transformation. They illustrated this point by referencing the impact of machines on domestic labor, providing an example of how technology (eg, washing machines) historically made life easier.

Participants highlighted the positive impact of technology in assisting them, that is, a “bonus of technology.” For instance, they mentioned services that identify hearing difficulties and use voice recognition technology. This included technologies such as cameras in the homes of older adults that allow families to monitor and ensure the well-being of their older members, offering a sense of security; videoconferencing tools (eg, Zoom [Zoom Video Communications]), which were described as instrumental in reducing loneliness, enabling individuals to engage in physical exercises, socialize, and attend events remotely; and other technologies used by doctors to communicate medical information before appointments, saving travel time through web-based appointments and receiving text reminders for medical appointments.

Yet, and despite these positive aspects, technologies and digitalization of services more broadly were framed as currently inaccessible with unfulfilled potential, creating a contrast between the envisioned benefits and the realities experienced by participants (“There is technology, I’ve seen it, people who need it don’t get it”).

#### Lack of Interoperability in Services, Institutions, and Within the NHS

A prevailing theme revolved around failings in the implementation of digital services and systems, spanning functionality and interoperability between services and departments, which leads to further complications when accessing essential services (“She could not see my account details”). On the one hand, this questions the effectiveness of urging technology use while facing ineffective implementation (“if the surgery can’t do it, what hope do you have”; “The GP/health services are telling us to use the services, but they don’t use them themselves. They send text messages we cannot even reply to and ask us to go on a website, but we are missing out on services”). On the other hand, it draws attention to the very design of these services, which are perceived as non–user-friendly, challenging to navigate, characterized by complex interfaces, and lacking in direct communication options such as telephone numbers and one-to-one interactions (“DWP [Department for Work and Pensions], this is actually serious and it’s threatening, and I cannot even call them out...when they call their number comes up as unknown and you have to wait for weeks and weeks”). Relatedly, text messages sent by the GP or the NHS system lacking reply options and instructions to visit websites posed challenges, especially for older individuals.

This lack of interoperability was often intertwined with intricate and dysfunctional bureaucratic processes within institutions. Participants were critical of the inflexibility of the systems in place, noting that deviating from what is perceived as the “right answer” could lead to requests being ignored or deemed invalid. This was particularly challenging for individuals with dyslexia, as the questions posed were difficult, and selecting the correct response became a hurdle. In addition, participants shared accounts of malfunctions and the necessity for prolonged phone calls, sometimes lasting up to an hour, especially in cases where individuals lacked smartphones (“And if you call you end up on the phone for hours because you don’t have a smartphone”).

Interestingly, we observed a relationship between poor functionality and lack of interoperability, whereby they can exacerbate each other, and preexisting difficulties, together. For example, NHS digital systems were described as “difficult” and “surreal.” One participant commented, “How many people really do this?” and that “Every new app makes my brain shudder.” They voiced confusion over accessing health care via various methods, leading to uncertainty (“I don’t know whether I am using the right thing”). In many cases, this results in challenges when individuals need to navigate multiple web pages or hop between apps, especially when the individual has multiple responsibilities (eg, caring for a child and a parent). Mostly, these issues lead to significant delays (“The nurse can’t help me”; “Two weeks from chemist to doctor, online 1-to-1 consultancy and still cannot get my medication”), but they also exacerbate carers’ lack of agency and highlight power imbalances. For example, for benefits claimants, participants expressed wariness and suggested that the forms and processes were designed to be confusing, so individuals in need of financial support would not be able to apply to claim it (“you can easily think that doesn’t apply to me”).

#### Research Coproduction for Reclaiming Agency

Throughout conversations, participants highlighted the need for robust carer representation, specifically, including individuals from underrepresented backgrounds in the creation of policy more broadly and digital “solutions” more specifically. They specifically discussed that policy making needs to be based on engagement and collaboration with diverse carers, in ways that acknowledge their lived experience of caring for and accessing care. Volunteers at carers’ forums and parent carers were deemed essential, and participants emphasized the need for continuous listening to carers at every stage of policy making. They expressed frustration with policies that have been drafted for them but without them (ie, no consultation beforehand) because this has led to initiatives that are not helpful nor needed (“it’s like going to the hotel...it says it’s disabled friendly and it’s absolutely not”).

The same principles underpinned conversations relating to technology design and designers and underlined the importance of co-designing with prospective users of technology rather than merely for them. Interestingly, these elements were considered in terms of designing and testing, as well as advertising, so that features and promises are aligned with reality and factual needs.

In short, collaboration with carers was constructed as essential for effective interventions, and policy makers were urged to invest in research on digital inclusion, following a holistic approach that values the input and experiences of carers in shaping policies and technology solutions. In doing so, one interesting finding was that participants highlighted that more coproduced qualitative research is needed, as this allows them to reclaim their agency and communicate their needs clearly, and this was contrasted to surveys, which are often closed-ended, leaving little room to properly engage with lived experiences.

## Discussion

### Principal Findings

In this study, we focused on the implications of the digitalization of social care on unpaid carers. Our aim was to explicate the perceptions and the lived experience of unpaid older carers, in particular, concerning digitalization, with the view to unpack and theorize around the implications of accelerated digitalization within a highly sensitive domain.

Our findings indicate that individuals who provide or receive care currently experience being “pushed” on the web, whereby they feel as if they are being coerced to access care services digitally, with limited alternative options. Their experiences are far removed from the ideal presented in the Department for Health and Social Care and the NHS’s “A Plan for Digital Health and Social Care” [12]:

Sarah can choose a telephone, video, or in-person meeting for her first IAPT appointment, during which she will be assessed. During her video assessment, Sarah chooses a clinician-guided digital therapy app that she can log onto in her own time, day or night, and is easy to use as it is interactive. She can also choose to have her sessions in her preferred language via chat, audio or video.

12

The pressure to use web-based services to access support is met with not only a barrier in terms of the will or motivation of unpaid carers to do so but also a lack of meaningful support to enable this in practice. Such support would take the form of digital skills training, including privacy and safety, as well as service design that is accessible and free of ableist assumptions. Currently, however, our insights suggest that such support is considerably lacking, which results in what we term the “great digital disconnect”: a situation whereby the digitalization interventions and programs seem to ignore, by omission or commission, those exposed to digital poverty and who are most likely to need social care support, that is, the disconnect between goals and actuality. However, this “disconnect” in service design is not an entirely new concept. For example, Stirling and Burgess [29], when examining smart telecare provision, uncovered disparities between intended goals and actual outcomes due to challenges arising from organizational and operational complexities. We extend this by arguing that such disparities may also exist due to ignorance of or indifference toward the contextual conditions of those who engage with the social care system. It is noteworthy that the described constructions and participants’ experiences, characterized by significant waiting times and dehumanizing conditions, resonate with Weber’s [30] notion of bureaucracy as an “iron cage” where individuals are subjected to challenging and counterproductive rules, leading to dehumanization and dysfunction within organizational structures, ultimately disregarding the needs of those seeking assistance.

Our findings indicate that older, unpaid carers wish to resist prevalent assumptions of universal access very clearly that portray everyone, irrespective of circumstances and choices, as being willing and able to access web-based services. This is congruent with earlier research. Choudrie et al [31], for example, found that older adults, in many cases, are willing and able to join the digital world, yet they do not do so because the available services do not offer any added value in their everyday lives. Similarly, Wyatt [32] criticized such universal assumptions regarding access and usefulness and went further on to highlight that nonusers’ situations and needs should also be factored in when thinking about technology design.

Our findings echo the aforementioned findings but further explain that such assumptions and the accelerated replacement of physical solutions with digital ones lead to a power imbalance between the state and beneficiaries, whereby unpaid carers are left with very few choices and little or no alternatives. This results in pseudoconsent and coercion and reinforced exclusions, where individuals experience powerlessness in sharing their data. Lutz et al [33] described this as privacy cynicism, and indeed, data capitalism involves the extraction of personal data for extensive monetization, presenting users—in this case unpaid carers—with dilemmas between privacy and social connection or accessing services and benefits. Therefore, the digitalization of social care becomes ideologically dilemmatic [34] where older carers provide superficial consent, leading to passivity not only due to full acceptance of dominant ideologies but also due to a lack of effective dissent expression [28]. Maglaras [35] noted limited resistance possibilities due to the dominance of the ruling class’s ideology. In this respect, we argue that unpaid carers’ experiences illustrate the increasing dominance of digitalization and the ways imposed digitalization links to what is called “surveillance realism” [36], whereby unpaid carers simply resign or give up, rather than truly consent in terms of parting with their data. However, there is still the possibility of recognizing the potential of alternative solutions—although rendered practically impossible—suggesting the digitalization of care has not yet reached a common sense status [37] or developed complete “ideological power” [38].

Against this background, we wish to highlight the implications of surveillance realism, unpaid carers’ resignations, and the neoliberalization of social care. Participants criticized the paradoxical relationship between the lack of services and social care support and the efficiencies and savings unpaid caring produces for the state. On the one hand, they challenged the notion of being solely responsible for acquiring equipment and broadband connections, which is reminiscent of the neoliberal ideals of individual responsibility [28,39], which see the individual solely responsible for their welfare. Participants highlighted the inadequacy of governmental benefits–related funding, echoing societal norms within neoliberalism that valorize volunteering and unpaid labor while deeming social care too costly for the market. On the other hand, however, power imbalances and dominant market logics lead unpaid carers to resign, adopt the same logic, and position themselves as idealized neoliberal individuals, framing their caregiving labor as a form of capital production that benefits the government. To an extent, this reflects the erosion of the welfare state under neoliberalism, where volunteering and unpaid labor become normalized to facilitate private service delivery [40]. In this respect, our study highlights how, under surveillance realism and digital capitalism, unpaid carers adopt market-centric logic to make sense of their experience and as a form of negotiating their worth and greater access to social care.

In this context, we introduce alienation to theorize the digital exclusion experienced by carers. Alienation manifests as a disconnection from one’s agency, resulting in a sense of estrangement from various aspects of life, including nature, labor, and social connections. Baines et al [41] discussed alienation of the social care worker under capitalistic constraints, but our study shows that unpaid carers, too, are constrained by a production system where they are treated as instruments rather than recognized as social beings, perpetuating a cycle of alienation from their labor and potential as human beings. In digital societies, production chains aim to shape individuals, further exacerbating this alienation [42].

### Limitations

We note that our study comes with certain limitations. However, the implications of the digitalization of social care can vary depending on the sociodemographics of the unpaid carer, such as sexual identity, race, disability, and socioeconomic status [20]. As such, these implications need to be further examined through an intersectional lens to uncover a more nuanced understanding. With regard to the methods used, our participants commented on the use of the story completion material and the inclusive nature we adopted in preparing them, as they were able to “see” themselves in these stories and identify with the developed personas without necessarily sharing personal details openly. Participants valued our method as it allowed them to distance themselves from the points raised during discussions. Yet, they also expressed that they would have liked for the activities to be longer (each focus group lasted about 2 hours) as they felt they had more to share with the group.

### Conclusions

Over recent years, in England and elsewhere, more social care services are becoming increasingly digital or moving to web-based provision, often framed as a means to increase the capacity of the social care system and create necessary efficiencies [10]. Digitalization is also aligned in policy discourse with offering people greater choice, control, power, and agency. While this is certainly possible, often, the digitalization of social care services risks excluding and disempowering those in need of support. Our findings and the concerns raised regarding the complexity of the technology and its exclusionary nature were often communicated with emotional language that conveyed a sense of abandonment. In addition, regarding the untapped potential of digitalization, while advantages were recognized, participants often felt let down as their needs were overlooked and stressed the importance of having a greater say in shaping technological solutions to ensure that these actually deliver on their potential benefits. Therefore, we highlight the importance of involving caregivers in the design of policies and technologies, emphasizing the need for research informed by lived experience and based on coproduction to allow for the meaningful integration of expertise and experience and for enabling person-centered care [43]. This can further enhance accessibility and usability by design but also requires challenging entrenched habits and assumptions [44].

## Appendix

Multimedia Appendix 1 Coding structure.

## Abbreviations

GP: general practitioner
NHS: National Health Service
T&Cs: terms and conditions
