# Supplementary material for: The Digitalization of Social Care in England and Implications for Older, Unpaid Carers: Constructionist Thematic Analysis
Source: J Med Internet Res. 2024 Oct 24;26:e60056. doi: 10.2196/60056 (PMC11544341; doi:10.2196/60056)
Supplement: Multimedia Appendix 1 [file jmir_v26i1e60056_app1.docx]

## Multimedia Appendix

**Table S1.** Coding Structure

| **Themes** | **Subthemes** | **Representative Extracts** |
| --- | --- | --- |
| Being pushed online | Lack of agency and pseudo-consent | *"I want people to have an alternative"*  *“Deliberately written to be non-informative!”*  *“You have to agree, or you don’t get the services.”*  *“These documents are a legal contract, meaning they can't get the blame for anything.”* |
|  | Maintaining alternatives | *“Not everybody wants to learn”*  *“I’ve lived without that my whole life, I don’t need this”.*  *"We have to be inclusive, we can't make people do it,"* |
|  | Digital alienation and exclusion | *"Everything is horrible”*  *"Nobody cares"*  *“Technology is amazing but it excludes so many people.”*  *"Nobody cares about me."*  *“People are not kind to older people."* |
| Supporting carers to digitalise | Skills gap and training needs | *“I felt very guilty and I still do” (for lacking technical knowledge)*  *“We lack knowledge”. "I don't know how to download an app (…) people do it for me”.* |
|  | Concerns regarding privacy and surveillance | *“You give all your personal information”*  *"How secure is all of this?"*  *“People are worried about where data go and what happens to their information”*  *“I don’t think I am stupid and I almost lost all my money. Called a mate and helped me out at the very last minute. It could happen to everybody.”* |
|  | Issues of Ableism and Accessibility | *"There are things but… how do we access them."*  *"Access is important... we need accessible things because it is good stuff, but what’s the point if it's not accessible?".*  *"You are given something you forget how to do it, and you feel embarrassed."*  *"My dyslexia makes me panic when things come through."* |
| The great digital disconnect | Lack of connectivity | *"What should take you one hour takes you all day."*  *"I tried that 4-5 times, and I rang because it wasn’t working… I gave up on it."*  *"If you have a slow internet connection, you have to start over again (...) frustrating and not helpful."* |
|  | Cost of connectivity | *“Lots of money for crap”*  *“Broadband services are a cutthroat business”.*  *“Nightmare”*  *“I was paying a lot having to do things.”* |
|  | Idealised neoliberal subjects | *“What do you do about that... tablets, free SIM cards, other things."*  *"Give them money, give them laptops."*  *“In the end the more savvy we are the more money we save”*  *“We are worth investment”*  *“We save the government millions of pounds …the stress we are under, anxiety”* |
| The unfulfilled potential of digitalisation | Benefits of technology and the unfulfilled potential of digitalisation | *“There is technology, I’ve seen it, people who need it don’t get it”.* |
|  | Lack of interoperability in services, institutions and within the NHS | *"And if you call you end up on the phone for hours because you don't have a smartphone."*  *"DWP (Department for Work and Pensions) this is actually serious and it's threatening, and I cannot even call them out then when they call their number comes up as unknown and you have to wait for weeks and weeks."* |
|  | research co-production for reclaiming agency | *“It's like going to the hotel it says its disabled friendly and it's absolutely not”* |
